# Spatiotemporal Pattern of Doublecortin Expression in the Retina of the Sea Lamprey

**DOI:** 10.3389/fnana.2016.00005

**Published:** 2016-01-29

**Authors:** Blanca Fernández-López, Daniel Romaus-Sanjurjo, Pablo Senra-Martínez, Ramón Anadón, Antón Barreiro-Iglesias, María Celina Rodicio

**Affiliations:** Faculty of Biology, Department of Cell Biology and Ecology, CIBUS, Universidade de Santiago de CompostelaSantiago de Compostela, Spain

**Keywords:** agnathans, DCX, acetylated α-tubulin, cytokeratin, retinal development, Müller cells, retinal pigment epithelium

## Abstract

Despite the importance of doublecortin (DCX) for the development of the nervous system, its expression in the retina of most vertebrates is still unknown. The key phylogenetic position of lampreys, together with their complex life cycle, with a long blind larval stage and an active predator adult stage, makes them an interesting model to study retinal development. Here, we studied the spatiotemporal pattern of expression of DCX in the retina of the sea lamprey. In order to characterize the DCX expressing structures, the expression of acetylated α-tubulin (a neuronal marker) and cytokeratins (glial marker) was also analyzed. Tract-tracing methods were used to label ganglion cells. DCX immunoreactivity appeared initially in photoreceptors, ganglion cells and in fibers of the prolarval retina. In larvae smaller than 100 mm, DCX expression was observed in photoreceptors, in cells located in the inner nuclear and inner plexiform layers (IPLs) and in fibers coursing in the nuclear and IPLs, and in the optic nerve (ON). In retinas of premetamorphic and metamorphic larvae, DCX immunoreactivity was also observed in radially oriented cells and fibers and in a layer of cells located in the outer part of the inner neuroblastic layer (INbL) of the lateral retina. Photoreceptors and fibers ending in the outer limitans membrane (OLM) showed DCX expression in adults. Some retinal pigment epithelium cells were also DCX immunoreactive. Immunofluorescence for α-tubulin in premetamorphic larvae showed coexpression in most of the DCX immunoreactive structures. No cells/fibers were found showing DCX and cytokeratins colocalization. The perikaryon of mature ganglion cells is DCX negative. The expression of DCX in sea lamprey retinas suggests that it could play roles in the migration of cells that differentiate in the metamorphosis, in the establishment of connections of ganglion cells and in the development of photoreceptors. Our results also suggest that the radial glia and retinal pigment epithelium cells of lampreys are neurogenic. Comparison of our observations with those reported in gnathostomes reveals similarities and interesting differences probably due to the peculiar development of the sea lamprey retina.

## Introduction

Doublecortin (DCX) is a member of the family of DCX proteins, a group of microtubule-associated proteins (for a review, see Dijkmans et al., 2010) characterized by the presence of a microtubule-binding domain (the DCX domain) that stabilizes microtubules and causes bundling (Kim et al., 2003). Identified by its mutation in the human X-linked lissencephaly and “double cortex” syndrome (Gleeson et al., 1998), DCX is generally expressed in immature and migrating neurons (Francis et al., 1999; Friocourt et al., 2003) and it appears to be involved in neuronal differentiation and migration. DCX is also expressed in differentiated neurons, where it has been suggested to play a role in processes such as neuronal plasticity, axonal outgrowth or synaptogenesis (Nacher et al., 2001; Brown et al., 2003; Yang et al., 2004; Capes-Davis et al., 2005). DCX has been considered a marker for newly generated neurons because most DCX-positive cells express early neuronal antigens (Rao and Shetty, 2004). However, DCX expression in cells expressing the oligodendrocyte precursor marker Olig2 has been recently reported (Diaz et al., 2013).

In the retina, DCX expression has been investigated in mammals, chick, in the short-lived teleost *Nothobranchius furzeri* and in sharks. In the developing mouse retina, the DCX transcript is expressed in the postmitotic inner neuroblastic layer (INbL) and in radially arranged cells in the outer neuroblastic layer (ONbL; Reiner et al., 2006). Interestingly, the retina of DCX mutant mice shows normal layering (Corbo et al., 2002). In the rat retina, DCX protein is highly expressed during the embryonic period, first in radially orientated cells in the mantle zone and then in cells of the inner part of the retina and the middle of the neuroblastic layer, decreasing its expression during the postnatal period (Lee et al., 2003); although, DCX expression has been observed in horizontal cells of retina in the adult rat (Wakabayashi et al., 2008). In the chick, DCX expression has been reported in neural progenitors in early embryonic stages and in developing ganglion cells and horizontal cells in later stages of development (Kim and Sun, 2012). In young adults of *Nothobranchius furzeri*, DCX expression has been reported in neuronal processes juxtaposed to the germinal margins of active neurogenesis (Tozzini et al., 2012). In a shark retina, DCX is initially expressed in immature cells and cell processes before retinal layering and later on, in ganglion, amacrine, bipolar and horizontal cells, with a pattern that is maintained in juvenile and adult sharks (Sánchez-Farías and Candal, 2015).

Lampreys are living representatives of the most ancient lineage of vertebrates, the agnathans or cyclostomes. The key phylogenetic position of lampreys, together with their unusual life cycle makes them an interesting “evo-devo” animal model. Lamprey eyes exhibit distinct larval and adult stages with highly different characteristics. The rudimentary eyes of larval lampreys are under a thick, nontransparent skin and are not image-forming eyes (Kleerekoper, 1972), but simple photoreceptive organs like an ocellus (Villar-Cerviño et al., 2006); while the eyes of adults are adapted to a well-developed vision. Accordingly, the retina of lampreys follows a bimodal pattern of development. The formation of the lamprey retina begins in embryogenesis, and at the end of the prolarval stage it consists of a retinal pigment epithelium that shows melanin granules and the sensory retina. The latter consists of two layers of cells, the inner region with ganglion cells and the outer region with photoreceptors, with very few bipolar cells between them (Meléndez-Ferro et al., 2002). In early larvae the small central retina, which consists of a single type of photoreceptor as well as bipolar, ganglion and Müller cells, is flanked by a marginal proliferating zone (de Miguel and Anadón, 1987; Rubinson and Cain, 1989; Meléndez-Ferro et al., 2002; Villar-Cerviño et al., 2006; Villar-Cheda et al., 2006). The differentiated retina grows slowly during larval stages until metamorphosis, which occurs several years after hatching (Meléndez-Ferro et al., 2002). Interestingly, during the larval period horizontal or amacrine cells are not neurochemically differentiated (Villar-Cerviño et al., 2006; Abalo et al., 2008). During the second half of the larval period, a largely undifferentiated zone, the lateral retina, develops around the central retina. In large premetamorphic larvae, the lateral retina occupies more than 99% of the retinal surface, which remains largely neuroblastic and only ganglion cells appear differentiated in this region (de Miguel et al., 1989; Villar-Cerviño et al., 2006).

Metamorphosis in lampreys involves profound transformations in the lateral retina, which are similar to the changes that take place during retinal embryogenesis in other vertebrates, although it occurs after several years of larval life. During metamorphosis proliferation stops and the retina undergoes morphological and neurochemical maturation and gradual acquisition of the layered appearance characteristic of the adult retina. In early metamorphic lampreys (stages M1–M2) the marginal neuroepithelial region is not distinguishable (Villar-Cheda et al., 2008) and the neurochemical differentiation of the amacrine cells begins, while the differentiation of the horizontal cells (Abalo et al., 2008) and adult type photoreceptors (Villar-Cheda et al., 2008) starts in M3 individuals. The distinction between central and lateral retina is appreciable until late metamorphic stages (M6–M7). In the adult retina, most of ganglion cells are located between the inner nuclear layer (INL) and the inner plexiform layer (IPL) and only a few are located in the IPL (Fritzsch and Collin, 1990). The optic fiber layer is located in the outer IPL (Dalil et al., 1990; Fritzsch and Collin, 1990).

Despite the importance of DCX in neurodevelopment, its expression in the brain of basal vertebrates has not been investigated and little is known regarding the spatiotemporal pattern of expression of this protein in the vertebrate retina. Here, we employed immunocytochemical methods using an anti-DCX antibody to investigate its expression in the prolarval, larval and adult retinas of the sea lamprey. In order to characterize the DCX-expressing cells in premetamorphic retinas, we also used an anti-acetylated α-tubulin (α-tubulin) antibody and an anti-cytokeratin antibody (Merrick et al., 1995) as specific markers for neuronal cells and glial cells of lampreys, respectively. Moreover, we used tract-tracing methods to label mature ganglion cells. Characterization of retinal DCX expression in lampreys will provide valuable information about of the roles and evolutionary conservation of DCX patterns in the retina of vertebrates.

## Materials and Methods

### Animals

Pigmentation (P2 and P3), gill cleft (P4–P7) and burrowing (P10, P12, P16 and P20) prolarvae (*n* = 32), larvae (between 20 and 145 mm in body length, *n* = 20), metamorphic (M2, M4 and M6; *n* = 4) and young postmetamorphic (*n* = 6) and upstream migrating adults (*n* = 3) were used. Prolarvae were obtained by *in vitro* fertilization of eggs obtained from sexually mature adult lampreys caught in the River Ulla (Galicia, Northwestern Spain). Fertilized eggs were reared in the laboratory under appropriate conditions of darkness and temperature. Stages of prolarvae, and early larvae are indicated by their age (e.g., P7 indicates 7 days posthatching, and so on; in our laboratory hatching occurred 11–13 days after fertilization). To classify prolarvae, we also used the stages defined by Piavis (1971) for the sea lamprey: hatching (P0–1), pigmentation (P2–3), gill-cleft (P4–7) and burrowing (P8–23) stages. In addition, to classify metamorphic individuals, we used the stages M1–M7 defined by Youson and Potter (1979) in sea lamprey. Larval and metamorphic individuals were caught in the River Ulla (Galicia, Spain) and maintained in an aerated aquarium with river sediment until processing. Body length was used as an indirect measure of larval age (30-mm larvae are 1-year old, whereas larvae about 130 mm are aged between 5 and 7 years). Young postmetamorphic adults (about 160 mm in length) were reared in the laboratory from metamorphic larvae or captured in the River Ulla. Upstream migrating (prespawning) adults (about 650–700 mm in total length) were purchased to local fishermen and processed immediately. Samples were killed by an overdose of MS-222 (Sigma) before use. All experiments were performed according to European Union and Spanish regulations for the care and handling of animals in research and were approved by the bioethics committee at the University of Santiago de Compostela.

### DCX Immunofluorescence

For DCX immunofluorescence, prolarvae, heads of larvae and metamorphic individuals and eyes of postmetamorphic lampreys were fixed by immersion in 4% paraformaldehyde in 0.05 M Tris buffer pH 7.4 (TBS) for 5–6 h. The tissue was then rinsed in TBS, cryoprotected by overnight immersion in 30% sucrose in TBS, and then embedded in Tissue Tek (Sakura, Torrance, CA, USA), frozen and cut in transverse planes on a cryostat (16–25-μm thick). Sections were incubated with a rabbit polyclonal antibody against DCX (Cell Signalling Technology; code no. 4604; dilution 1:300; immunogen: synthetic peptide [KLH-coupled] corresponding to human DCX), overnight at room temperature. A goat anti-rabbit immunoglobulin (Ig) antibody (diluted 1:200; Chemicon, Temecula, CA, USA) coupled to Cy3 was then applied for 1 h. The antibodies were always diluted with TBS containing 15% normal goat serum and 0.2% Triton X-100.

### Double DCX and α-Tubulin Immunofluorescence

Some premetamorphic (more than 100 mm in body length) larval heads were analyzed by double immunostaining against DCX and α-tubulin. Sections were first incubated overnight with a cocktail of the rabbit anti-DCX antibody and a mouse monoclonal α-tubulin antibody (Sigma, St. Louis, MO, USA; code no. T6793; lot. no. 6-11B-1, diluted 1:500; immunogen: tubulin from the outer arm of cilia of *Strongylocentrotus purpuratus*). The secondary antibody used for the anti-α-tubulin antibody was a goat anti-mouse IgG antibody coupled to fluorescein isothiocyanate (FITC; diluted 1:50; Chemicon, Temecula, CA, USA) combined with the same anti-rabbit Ig antibody used for single DCX labeling. Sections were incubated with the secondary antibodies for 1 h. All antibodies were diluted with TBS containing 15% normal goat serum, 10% normal swine serum, and 0.2% Triton X-100.

### Cytokeratin Immunohistochemistry

Some prolarvae (*n* = 4), larvae (*n* = 10) and postmetamorphic (*n* = 3) animals were analyzed by single immunostaining against cytokeratin. Prolarvae, larval heads and the eyes of postmetamorphic larvae were fixed by immersion in Clarke’s fixative for 2–3 h at room temperature and embedded in paraffin wax. Sections (10–14 μm thick) were cut on a rotary microtome. Dewaxed sections were incubated first with a mouse monoclonal anti-lamprey cytokeratin (LCM29) antibody (diluted 1:100; supplied by Dr. Selzer, Temple University, PA, USA). Then, sections were incubated with either a goat anti-mouse biotinylated immunoglobulin (diluted 1:500; Dako, Glostrup, Denmark) or an HRP-conjugated goat anti-mouse immunoglobulin (diluted 1:200; Bio-Rad, Hercules, CA, USA). All antibodies were diluted in TBS containing 15% normal goat serum and 0.2% Triton X-100. The sections incubated with the biotinylated secondary antibody were incubated with the avidin-biotin complex (Vector, Burlingame, CA, USA) for 30 min. The immunostaining was developed with 0.6 mg/ml 3-3-diaminobenzidine (DAB; Sigma) and 0.003% H_2_O_2_.

### Antibodies Specificity

The anti-DCX antibody was obtained against a peptide of the human protein, and the specificity was tested in rat brain extracts with western blot. Two bands of about around 45 kDa (DCX molecular weight) were observed, which were interpreted as a posttranscriptional modification of DCX (Francis et al., 1999). Western blots carried out with brain protein extracts of sea lampreys, have also shown two bands around 45 kDa (Barreiro-Iglesias et al., 2011).

The anti-α-tubulin antibody has been used as a cilia marker (Piperno and Fuller, 1985) and has also been shown to label neurons and their processes in the nervous system (Chitnis and Kuwada, 1990; Wilson et al., 1990). This antibody has been widely used as a neuronal cell marker (both fibers and perikarya) of both central and peripheral nervous systems in lampreys (Kuratani et al., 1997; Barreiro-Iglesias et al., 2008a,b,c, 2009).

The mouse monoclonal antibody against sea lamprey glial cytokeratin (LCM 29) has been previously characterized (Merrick et al., 1995) and used in studies about the spinal cord of sea lamprey (Lurie et al., 1994; Uematsu et al., 2004; Vidal Pizarro et al., 2004; Fernández-López et al., 2014).

As a control for secondary antibodies, the primary antibodies were omitted from some sections during the procedures. None of these control sections exhibited positive staining.

### Retrograde Tracing of Ganglion Cells

In order to label retinal ganglion cells, the tracer Neurobiotin (NB; Vector) was applied to the optic nerve (ON) exit in eyes of premetamorphic larvae (*n* = 3) and early metamorphic individuals (M2, *n* = 1). The eyes were left for 1 day at 4°C, with appropriate aeration conditions, in lamprey Ringer’s solution of the following composition (in mM): 137 NaCl, 2.9 KCl, 2.1 CaCl_2_, 2 HEPES. Then the eyes were fixed and processed for DCX immunofluorescence as above. For the detection of NB, the sections were incubated at room temperature with FITC-coupled avidin D (Vector) diluted 1:1000 in TBS containing 0.3% Triton X-100 for 3 h.

### Additional Material

Serial sections of prolarval and larval lamprey heads and of adult eyes stained with hematoxylin-eosin were at our disposal for anatomical comparison.

### Photography

Confocal photomicrographs were taken with spectral confocal laser microscopes (Leica TCS-SP2 or TCS-SP5). Bright-field photomicrographs were taken with a photomicroscope equipped with a DP-70 digital camera (Olympus). Photomicrographs were adjusted for brightness and contrast with Adobe Photoshop CS4 software.

## Results

**Figure 1:**
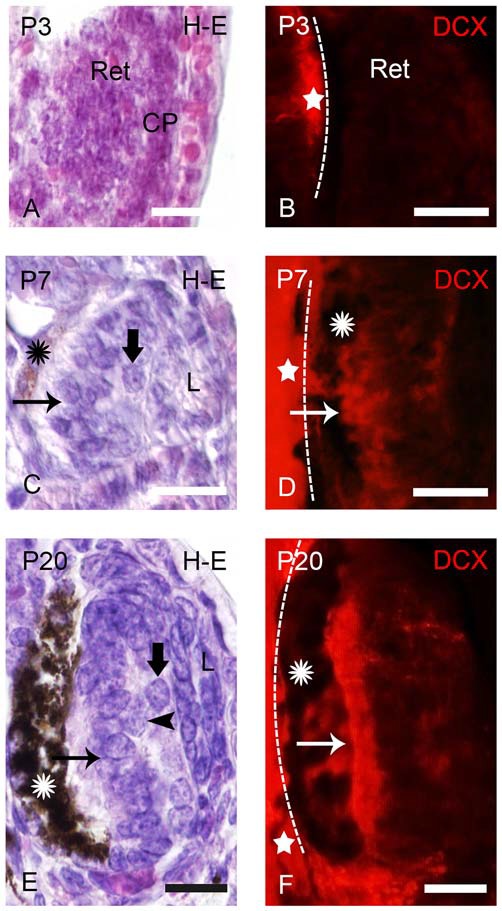
**Photomicrographs of transverse sections of retina in prolarval sea lampreys showing hematoxylin-eosine (H-E) labeling (A,C,E) or doublecortin (DCX) immunoreactivity (B,D,F).** The H-E and DCX panels are from different animals, but at similar levels. In the DCX panels the dashed lines indicate the limit between the retina and the brain (the brain is always to the left). **(A)** Retina of a pigmentation prolarva (a p3) showing the early retina (Ret), which is formed by undifferentiated cells. Note the presence of the crystalline placode at this developmental stage (CP). **(B)** Photomicrograph of the retina of a p3 prolarva showing the absence of DCX immunoreactivity. Note DCX immunoreactivity in the brain (star) in the same section. **(C)** Retina of a gill cleft prolarva (p7) showing the primordium of the lens (L), ganglion cells (thick arrow), photoreceptors (arrow) and the retinal pigment epithelium with melanin granules (asterisk). **(D)** DCX immunoreactivity in cells of the outer (photoreceptors) and inner layers of the retina of a p7 prolarva. **(E)** Retina of a burrowing prolarva (p20) showing ganglion cells (thick arrow), bipolar cells (arrowheads) and photoreceptors (arrow). The retinal pigment epithelium contains abundant melanin granules. **(F)** Retina of a p20 prolarva showing DCX immunoreactivity in perikarya and outer segments of photoreceptors and in a few cells and fibers of the inner region. In all photomicrographs dorsal is up and medial is to the left. Scale bars: 25 μm.

**Figure 2:**
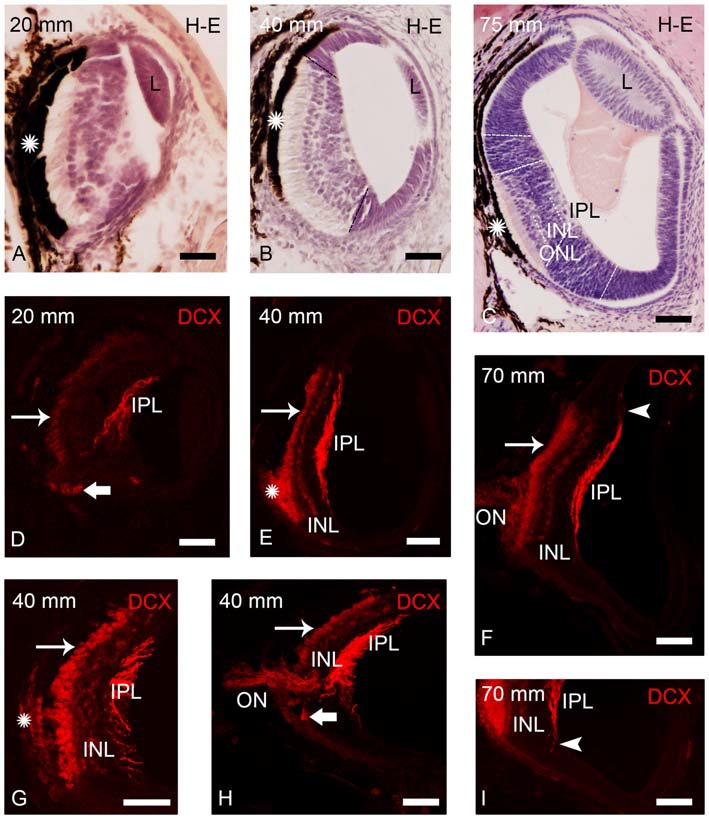
**Photomicrographs of transverse sections of retina of small larval lampreys stained with H-E (A–C) or immunostained for DCX (D–I). (A)** Retina of an early larva (20 mm), in which only the central retina is present. Note also the presence of the retinal pigment epithelium (asterisk) and the crystalline lens (L). **(B)** Retina of a 40 mm larva, which shows the central and lateral retinas. **(C)** Central and lateral retinas in a larva of 75 mm. Dash lines mark the limit between the central and lateral retinas in **(B,C)** and also between the central and peripheral regions of the lateral retina in (**C**; see Villar-Cheda et al., 2008). Dotted lines mark the limits between layers in the central retina in **(C)**. **(D)** DCX immunoreactivity is observed in the perikarya of photoreceptors (thin arrow), in fibers of the inner plexiform layer (IPL) and in round cells located in the ventral retina, close to the exit of the optic fibers (thick arrow) in a 20 mm larva. **(E)** DCX immunoreactivity in a 40 mm larva. Expression is observed in the perikarya and the outer segments of photoreceptors, in fibers of the IPL and in cells of the outer layer of the inner nuclear layer (INL). The DCX-ir retinal pigment epithelium is indicated with an asterisk. **(F)** Retina of a 70 mm larva showing DCX immunoreactivity in the perikarya and outer segments of photoreceptors, in fibers of the optic nerve (ON) and in cells and fibers of the INL and IPL, respectively. **(G)** Detail of the central retina of a 40 mm larva. **(H)** Retina of a 40 mm larva showing DCX immunoreactivity in round cells of the ventral retina located close to the ON. **(I)** Detail of the retina of a 70 mm larva showing DCX-ir radial processes (arrowhead) in the boundary between the central and lateral retina. Note the absence of DCX immunoreactivity in the lateral retina. In all photomicrographs dorsal is up and medial is to the left. Scale bars: 25 μm in **(A,D,G,I)**; 50 μm in **(B,C,E,F,H)**.

**Figure 3:**
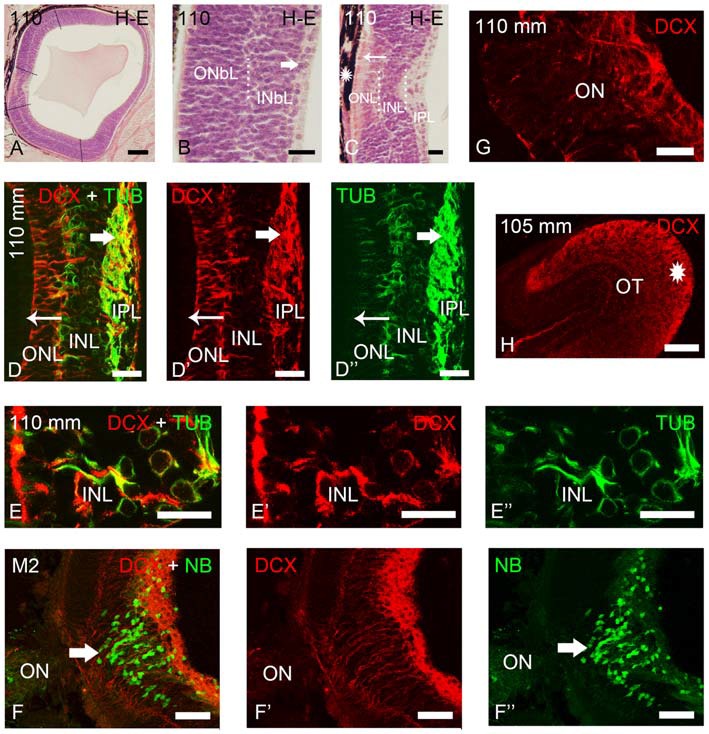
**Photomicrographs of transverse sections of retina and the optic tectum of premetamorphic and metamorphic larval lampreys stained with H-E (A–C) or immunostained for DCX, TUB and/or NB (D–H). (A)** Low magnification photomicrograph showing the central and lateral neural retina. Note that the lateral retina extends through most of the retina excepting the central region and a thin irideal retina. Dash lines mark the limit between the central and lateral retinas and between the central and peripheral portions of the lateral retina. **(B)** Detail of the central portion of the lateral retina showing distinguishable inner (INbL) and outer (ONbL) neuroblastic layers. A thin inner region with differentiated ganglion cells is also observed (thick arrow). **(C)** Detail of the central retina. The thin arrow points to photoreceptors. The asterisk indicates the dorsal retinal pigment epithelium bearing melanin granules. Dotted lines mark the limits between layers in the lateral retina in **(B)** and in the central retina in **(C)**. **(D–D″)** Double immunolabeling for DCX and α-tubulin in the central retina. Note the absence of DCX immunoreactivity in photoreceptors (thin arrow) and its presence in ganglion cells (thick arrow) and in cells of the INL. Note also the DCX/α-tubulin-ir double-labeled radial fibers coursing in the INL and ONL and DCX-ir/α-tubulin-negative end feet in the outer limitans membrane (OLM). **(E–E″)** Detail of the INL of the central retina of a premetamorphic larva showing the presence of DCX/α-tubulin-ir double-labeled cells. **(F–F″)** Absence of DCX immunoreactivity in the ganglion cells labeled after application of neurobiotin in the ON of a metamorphic larva (M2). **(G)** Detail showing DCX-ir fibers in the ON. **(H)** DCX-ir optic fibers in the optic tectum (asterisk). In all photomicrographs dorsal is up and medial is to the left. **(D–F)**: Overlay; **(D′–F′)**: DCX; **(D″,E″)**: α-tubulin; **(F″)**: NB. Scale bars = 75 μm in **(F–F″)**; 60 μm **(A)**; 50 μm **(H)**; 25 μm **(D–E″,G)**; 10 μm **(B,C)**.

**Figure 4:**
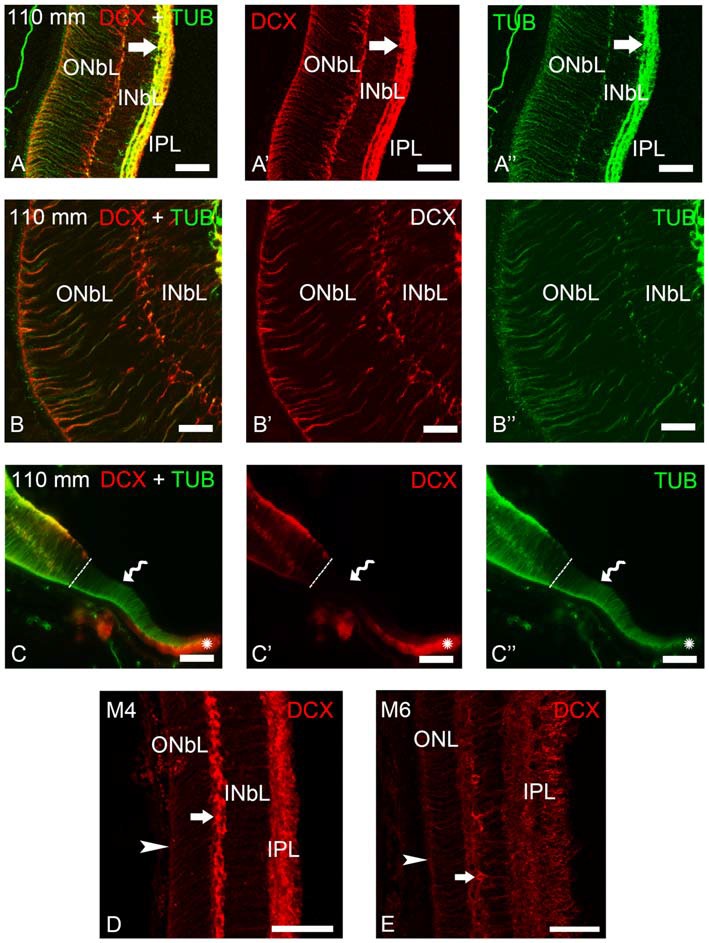
**Photomicrographs of transverse sections of the retina of premetamorphic and metamorphic larval sea lampreys showing anti-DCX and anti-α-tubulin immunoreactivities. (A–A″)** DCX/α-tubulin-ir double-labeled ganglion cells and fibers in the IPL and cells of the outer layer of the INbL in the lateral retina. Note the presence of double labeled radial fibers coursing throughout the neuroblastic layers and ending in the OLM. **(B–B″)** Detail of the lateral neural retina of a premetamorphic larva showing the presence of DCX/α-tubulin-ir double-labeled radial fibers in the INbL and ONbL. **(C–C″)** Photomicrograph of the border of the lateral neural retina of a premetamorphic larva showing the lack of DCX immunoreactivity in the growing irideal retina (curved arrow). Note that the outer epithelium of the irideal marginal region was DCX-ir (asterisks). Dash lines limit the marginal retina from the rest of the lateral retina. **(D)** In M4 larvae, DCX immunoreactivity was observed in fibers in the IPL, in radial fibers of the INbL and ONbL ending in the OLM (arrowhead) and in cells in the outer INbL (arrow). **(E)** In M6 larvae, DCX immunoreactivity was similar but retinal layers appear differentiated. **(A–C)**: Overlay; (**A′–C′**): DCX; (**A″–C″)**: α-tubulin. Scale bars: 50 μm **(A–A″,C–C″)**; 37.5 μm **(D,E)**; 25 μm **(B–B″)**.

**Figure 5:**
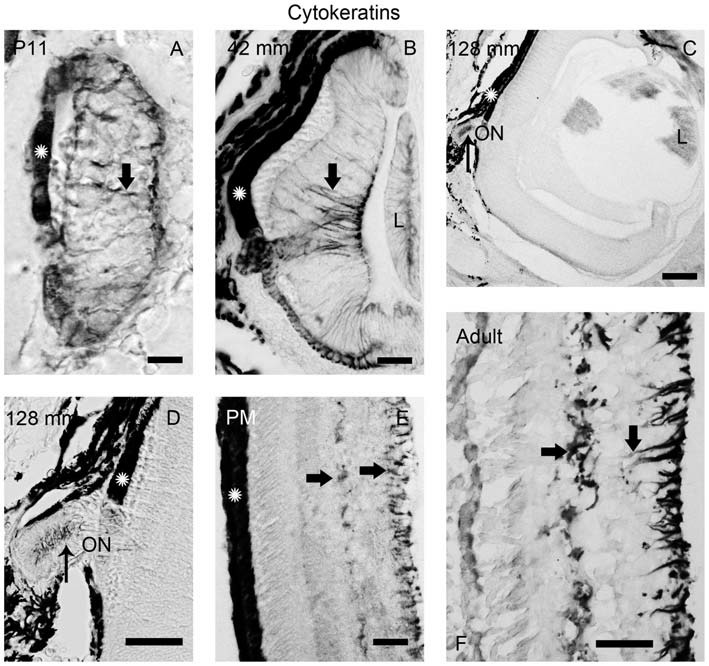
**Photomicrographs of transverse sections of the retina showing anti-cytokeratin immunoreactivity. (A)** Prolarval retina. **(B)** Retina of a small larva. **(C,D)** Retina of a premetamorphic larva **(C)** and detail of the ON **(D).** **(E,F)** Retina of postmetamorphic **(E)** and upstream migrating adult **(F)** sea lampreys. Cytokeratin-ir Müller cells (thick arrows) were observed in the prolarval **(A)**, small larval **(B)** and postmetamorphic/adult **(E,F)** retinas. Note the lack of cytokeratin labeling in the premetamorphic retina **(C)**, although the ON shows cytokeratin-ir astrocytes (thin arrows in **D**). In postmetamorphic/adult sea lampreys **(E,F)** only the inner processes of the Müller cells show anti-cytokeratin immunoreactivity. In all photomicrographs dorsal is up and medial is to the left. Scale bars: 12.5 μm in **(E)**; 25 μm in **(A,B,D)**; 50 μm in **(F)**; 100 μm in **(C)**.

**Figure 6:**
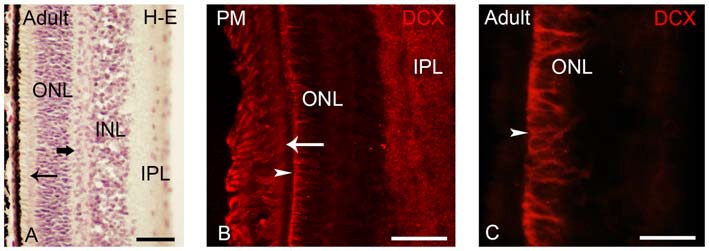
**(A)** Photomicrograph of a transverse section of the adult lamprey retina stained with H-E. The thick arrow points to horizontal cells in the outer part of the INL. The thin arrow points to photoreceptors. **(B,C)** Retinas of postmetamorphic **(B)** and upstream migrating **(A,C)** adults showing DCX immunoreactivity. In young postmetamorphic retinas **(B)** DCX immunoreactivity was observed in the outer segments of photoreceptors (thin arrow), in fibers in the IPL and in radial fibers of the ONL ending in the OLM (marked by an arrowhead). In upstream migrating adults DCX immunoreactivity was only observed in radial fibers coursing in the ONL and ending in the OLM **(C)**. In all larval photomicrographs, dorsal is up and medial is to the left. Scale bars: 25 μm in **(A)**; 50 μm in **(C)**; 75 μm in **(B)**.

DCX immunoreactivity was observed in the retinas of prolarvae from p5 onwards (Figure 1), larvae (Figures 2–5) and in young (postmetamorphic) and upstream migrating adults (Figures 5, 6). We observed variations in the expression pattern of DCX throughout the different developmental stages.

### DCX Expression in the Retina of Prolarvae

In “pigmentation” prolarvae (p2 and p3) the flattened eye cup consists of a retina with a thin medial (outer) wall and a thickened lateral (inner) wall (Figure 1A). The crystalline placode (CP) can be distinguished at this developmental stage (Figure 1A). No cells of the retina exhibited DCX immunoreactivity at this developmental stage, in spite of the presence of DCX expression in the brain (Figure 1B).

The eyes of “gill cleft” prolarvae (p4–p7) exhibited a lens primordium, the thick sensory retina and the retinal pigment epithelium with some melanin granules. The sensory retina is formed by two cell layers, an inner one with ganglion cells and an outer one with photoreceptors. The margin of the retina is formed by undifferentiated cells (Figure 1C). The first DCX expression was observed in p5 prolarvae. In the retina of these animals, DCX immunoreactivity was observed in most cells of the outer region and in some cells of the inner region (Figure 1D).

In burrowing prolarvae (p8–p23), the melanin granules in the retinal pigment epithelium are abundant. In the sensory retina, ganglion cells, photoreceptors and some bipolar cells could be distinguished (Figure 1E). In the outer region of the retina, DCX immunoreactivity was observed in the perikarya and outer segments of photoreceptors and in a few cells and fibers coursing in the inner region (Figure 1F).

### DCX Expression in the Retina of Larvae Under 100 mm in Body Length

In early larvae only the central retina is present (Figure 2A). As the development progresses the formation of the lateral retina begins (Figures 2B,C; de Miguel and Anadón, 1987). Until premetamorphic stages, DCX immunoreactivity was only observed in the central retina of larvae (Figures 2D–I). In all larvae smaller than 100 mm in body length, DCX immunoreactivity was observed in the perikaryon of photoreceptors (Figures 2D–H). In the small region ventral to the exit of the ON, the retinal pigment epithelium lacks melanin and thus DCX immunoreactivity could be observed in the outer segments of the photoreceptors (Figures 2E–G) and in retinal epithelial cells (Figure 2G), whereas this could not be observed in the heavily pigmented dorsal region. Strong DCX immunoreactivity was also observed in optic fibers coursing in the IPL (Figures 2D–I) and in the ON (Figures 2F,H).

From larvae of 40 mm in body length, weak DCX immunoreactivity was also observed in a layer of cells located in the outer part of the INL (Figures 2E–I). In addition, in larvae less than 50 mm in body length, some DCX-ir round cells were observed in ventral retina very close to the exit of the optic fibers (Figures 2D,H). In larvae around 60–90 mm in body length, DCX immunoreactivity was also observed in radial processes located in the boundary between the central and lateral retina and coursing from the IPL to the outer limitans membrane (OLM; Figures 2F,I).

### DCX Expression in the Retina of Premetamorphic and Metamorphic Larvae

As previously shown by Villar-Cheda et al. (2008), the differentiated central retina of premetamorphic larvae is similar to that of younger larvae, but the lateral retina increased considerably in size in premetamorphic larvae, constituting most of the larval retina (Figure 3A). The most lateral retina is mainly neuroblastic although inner and outer sublayers became distinguishable in most central parts by the appearance of an intermediate region with a lower density of cells (Figure 3B). Ganglion cells are already differentiated in the innermost part of the INbL (Figure 3B; Meléndez-Ferro et al., 2002). In these larvae, photoreceptors are only present in the central retina (Figure 3C; Villar-Cheda et al., 2008). From M1 to M4 metamorphic stages, the organization of the retina is similar but the marginal zone is not distinguishable. In M5 larvae the outer segments of photoreceptors start to differentiate throughout the lateral retina and in late metamorphic larvae (M6–M7), as in young postmetamorphic adults, the distinction between central and lateral retinas is no longer possible.

In premetamorphic larvae, DCX immunoreactivity was observed in both the central and lateral retinas (Figures 3, 4). The expression of DCX in the central and lateral retina of M1–M4 metamorphic larvae was the same as in premetamorphic larvae. In the central retina photoreceptors do not show DCX immunoreactivity (Figures 3D–D″). DCX immunoreactivity was observed in some ganglion cells of the IPL (Figures 3D–D″) and in cells located in the INL (Figures 3D–E″). Although some DCX-ir cells of premetamorphic and metamorphic larvae are putative ganglion cells based on their location, the ganglion cells labeled after application of NB in the ON were not DCX-ir (Figures 3F–F″). DCX-ir fibers were observed in the IPL (Figures 3D–D″), ON (3G), and the layer of the fibers of the ON in the optic tectum (Figure 3H). DCX-ir radial processes were also observed coursing in the INL and ONL (Figures 3D–D″). Some of the radial processes formed end feet-like dilatations in the OLM (Figures 3D–D″).

Most of the lateral retina showed DCX immunoreactivity in ganglion cells of the IPL, in two rows of strongly labeled cells in the outermost INbL, in weakly labeled neuroblastic cells, in fibers of the IPL and in radial processes coursing through the neuroblastic layers, ending in the OLM (Figures 4A–B″). No DCX immunoreactivity was observed in the border of the lateral retina, while the retinal pigment epithelium of this marginal region was DCX-ir (Figures 4C–C″). In M4 metamorphic larvae, the labeled cells of the outermost INbL of the lateral retina became flattened tangentially strongly resembling horizontal cells (Figure 4D). In late metamorphic larvae (M6), in which there is no distinction between central and lateral retinas, in addition to radially oriented processes ending in the OLM and numerous fibers in the IPL, the tangentially flattened cells in the outer INL showed weaker DCX immunoreactivity (Figure 4E).

To determine the nature of the DCX-ir structures, double immunofluorescence methods were used for simultaneous detection of DCX and α-tubulin in the retina of premetamorphic larvae (Figures 3, 4). Both in the central and lateral retina, most of the DCX-ir perikarya were also α-tubulin-ir (Figures 3D–E″, 4A–C″). Most of the DCX-ir processes were also α-tubulin-ir (Figures 3D,E″, 4A–C″), although in some of them it was difficult to assess colocalization of the two proteins. Moreover, the DCX-ir endings of the radial fibers in the OLM of the central retina were α-tubulin-negative (Figures 3D–D″), although similar structures in the lateral retina were α-tubulin-ir and (Figures 4A–C″).

In order to investigate the possible glial nature of DCX-ir radial processes ending in the OLM, anti-cytokeratin immunohistochemistry was performed (Figure 5). Of note, no cytokeratin-ir Müller glial cells were observed in the retina of premetamorphic lampreys (Figures 5C,D). However, astrocytes in the ON of the same animals (Figures 5C,D) were cytokeratin-ir as well as the Müller cells in the central retina of late prolarvae (Figure 5A), small larvae (Figure 5B) and adults (Figures 5D,E). The presence of cytokeratin-ir astrocytes in premetamorphic larvae indicates that the absence of cytokeratin expression in the retina is not a false negative result.

### DCX Expression in the Retina of Young Postmetamorphic and Upstream Migrating Adult Lampreys

After metamorphosis the entire retina became differentiated, showing two types of photoreceptors and the distinction between central and lateral retina is not observed (Figure 6A; Villar-Cerviño et al., 2006; Villar-Cheda et al., 2008). In young postmetamorphic lampreys, DCX immunoreactivity was only observed in the outer segments of the photoreceptors, in fibers in the IPL and in radial fibers coursing in the ONL and ending in the OLM (Figure 6B). In the retina of upstream migrating adults, radial fibers coursing in the ONL and ending as end feet in the OLM (Figure 6C) showed DCX immunoreactivity.

## Discussion

### The Proliferating Regions in the Retina of Lampreys do not Express DCX Immunoreactivity

No DCX immunoreactivity was observed in the retina of pigmentation prolarvae, in the marginal retina of gill-cleft and burrowing prolarvae and in the lateral and marginal retinas of larvae under 100 mm in body length. All cells of these regions and at these stages show expression of PCNA, a reliable marker for cycling cells (Meléndez-Ferro et al., 2002; Villar-Cheda et al., 2008). Although in premetamorphic larvae DXC expression was observed in the lateral retina, its regional expression is not the same than that of PCNA as demonstrated by Villar-Cheda et al. (2008): the proliferative marginal region (Villar-Cheda et al., 2008) lacks DCX immunoreactivity (present results). These results indicate that the proliferating cells of the retina of the sea lamprey do not express DCX. DCX expression in proliferating cells of the retina has been reported in sharks (Sánchez-Farías and Candal, 2015), but not in teleosts (Tozzini et al., 2012), or in mammals (Lee et al., 2003). The absence of DCX in proliferating cells of the retina of lampreys suggests that its presence in proliferating cells of sharks is a derived condition.

### The Expression of DCX in the Lateral Retina of Premetamorphic Lampreys Resembles the Expression of DCX in the Early Retina of Other Vertebrates

A surprising result of our investigation is that the earliest cells expressing DCX in the retina of the sea lamprey are ganglion cells and photoreceptors in differentiation. This differs from the early development of the retina in rats (Lee et al., 2003) and sharks (Sánchez-Farías and Candal, 2015), in which radially orientated cells are the first ones showing DCX immunoreactivity. The simplified early development of the retina of larval lampreys, in which a small retina becomes differentiated during the prolarval stage, could explain this difference. Owing to the small size and simple organization of this retina, no cell migrations appear to occur. Like in the early retina of other vertebrates, the expanded immature lateral retina of premetamorphic larvae showed DCX expression in radially oriented cells. Most but all of these cells were also α-tubulin-ir, a marker of neuronal cells. These cells have been interpreted as migrating neuronal precursors (Lee et al., 2003; Sánchez-Farías and Candal, 2015). Accordingly, in lampreys DCX may also be involved in neuronal migration during retinal development, but whereas in other vertebrates this process takes place during the embryonic period, in lampreys it is delayed several years to later larval stages. These suggests that the role of DCX in the migration of retinal cells is a conserved trait that appeared early during the evolution of the vertebrate retina.

### Some Immature Retinal Neurons Express DCX

Immature DCX-ir cells were observed in the developing retina of the sea lamprey. These include immature ganglion cells and cells that by their position in the outer part of INL of the larval central retina, in the outer part of the INbL of the lateral retina of premetamorphic and early metamorphic larvae and in the outer INL of late metamorphic larvae could correspond to immature horizontal cells. DCX-ir immature ganglion and horizontal cells are observed in the developing retina of other vertebrates (Lee et al., 2003; Kim and Sun, 2012; Sánchez-Farías and Candal, 2015) suggesting that this feature appeared early and has been conserved in vertebrate evolution. In contrast to mature horizontal cells of lampreys, those of rats (Wakabayashi et al., 2008) and sharks (Sánchez-Farías and Candal, 2015) show DCX expression. The adult expression of DCX in horizontal cells could then be a novel character of jawed vertebrates.

In lampreys, DCX immunoreactivity is lost in perikarya of ganglion cells whose axons have reached the ON, although their processes in the IPL, in the ON and optic tectum are DCX-ir. In the sea lamprey, the optic tectum matures during the metamorphosis (de Miguel and Anadón, 1987). So, the late expression of DCX in the processes of ganglion cells could be related to the formation of neuronal process and the establishment of synaptic circuitry. A role for DCX in the reorganization of microtubules during the formation of new neurites and synaptogenesis in the IPL of the rat retina has been proposed (Lee et al., 2003). The horizontal cells of the sea lamprey retina differentiate immunohistochemically in middle metamorphic stages (Abalo et al., 2008) and they establish synaptic contacts with photoreceptors in late metamorphic stages (de Miguel, 1989; Villar-Cheda et al., 2008). So, DCX could be also playing a role in the formation of neurites and the establishment of synaptic connections in putative horizontal cells of lampreys. Present and previous results indicate that the role of DCX in the establishment of the retinal circuits is a conserved character in vertebrate evolution.

### Outer Segments of Photoreceptors of the Sea Lamprey showed DCX Immunoreactivity

We observed DCX expression in photoreceptors at different developmental stages. As far as we are aware, DCX expression has not been reported in photoreceptors of jawed vertebrates, although they express other members of the DCX family of proteins, RP1 and RP1L1. The expression of RP1 and RP1L1 proteins is high in the eye and low elsewhere in the body (Reiner et al., 2006) and they are involved in photoreceptor development (Conte et al., 2003; Liu et al., 2003, 2004). In lamprey, molecular weights of RP1 and RP1L1 proteins are about 240 and 200 kDa, respectively (Yamashita et al., 2009). However, our previous western blot results in lamprey with the present DCX antibody (Barreiro-Iglesias et al., 2011) indicate that no protein of these molecular weights is recognized by the anti-DCX antibody used in this study. The expression of DCX in photoreceptors could be a specialization of lampreys or the ancestral character that was lost in jawed vertebrates. The role of DCX in the development of lamprey photoreceptors deserves further investigation.

### DCX-ir Radial Fibers Ending as Endfeet in the OLM

In the lateral retina of lamprey larvae, strong DCX immunoreactivity was observed in two rows of cells located in the outermost part of the INbL. These cells exhibit radial processes ending as endfeet in the OLM. From their location and the morphology of endfeet, these cells are similar to radial glia (Müller cells), but instead of expressing cytokeratins, the proteins of the intermediate filaments of the glia of lampreys (Merrick et al., 1995), most of them express α-tubulin, which is considered a neuronal marker (Kuratani et al., 1997; Barreiro-Iglesias et al., 2008a,b,c, 2009). While they express DCX and α-tubulin as differentiating neurons, their morphology is alike Müller cells. In the larval central retina, similar DCX-ir endfeet were observed, but they did not express cytokeratins or α-tubulin. In mammals, comprehensive expression profiling techniques have shown that late progenitor cells and Müller cells are very similar at molecular level (for a review, see Jadhav et al., 2009). New studies are necessary to fully characterize these cells in lampreys.

The analysis of the expression of cytokeratins in the lamprey retina has also shown some surprising results. Expression of cytokeratins was observed in the Müller cells of the central retina in larvae, except in premetamorphic stages. This suggest that at this stage they experiment a process of dedifferentiation to retinal progenitor cells. In response to retinal damage in the postnatal chicken (Fischer and Reh, 2001) and in adult teleosts (Yurco and Cameron, 2005; Bernardos et al., 2007; Thummel et al., 2008; Lenkowski and Raymond, 2014), transient dedifferentiation of Müller cells to cells re-expressing retinal progenitor and stem cell markers occurs. In mammals, in response to a toxic injury, some Müller glial cells proliferate and produce a limited number of bipolar cells and rod photoreceptors that replace lost retinal neurons (Ooto et al., 2004).

On the other hand, Müller cells of both down and upstream migrating adult lampreys express cytokeratins in perikarya, horizontal processes and inner radial processes forming endfeet in the IPL, but not in outer radial processes ending in the OPL. Interestingly, we observed DCX immunoreactivity in endfeet in the OPL of the retina of both downstream and upstream migrant adults, therefore indicating that Müller cells of adult lampreys are polarized, with the outer processes showing some characteristics of radial glial cells. Since the marine phase of adult sea lampreys was not investigated, we do not know if these characteristics are maintained in growing adults. Our results would indicate that, in this basal vertebrate, the retinal Müller cells are more like to progenitor cells than in gnathostomes. In fact, Jadhav et al. (2009) have proposed that Müller glial cells are a form of late stage retinal progenitor cells that acquire some specialized glial function without irreversibly leaving the progenitor state.

### Immature Retinal Pigment Epithelial Cells Express DCX

Epithelial cells of the larval central retinal pigment epithelium (at least those ventrally located to the ON, which lack melanin), and those located in the irideal margin of the retina of premetamorphic and early metamorphic larvae showed DCX immunoreactivity. DCX expression has been also reported in cells of the retinal pigment epithelium of sharks (Sánchez-Farías and Candal, 2015) and in adult rat retinal pigment epithelium-derived cells that have neural morphology and express neural progenitor markers (Engelhardt et al., 2005). These cells have been interpreted as a progenitor population with potential for neuronal differentiation. These results suggest that DCX expression in the retinal pigment epithelium is a primitive feature in vertebrates, but if they are progenitor cells in lampreys needs to be investigated.

## Conclusion

Our results indicate that DCX plays a major role in the development of the neural retina of a representative of the oldest group of extant vertebrates, probably by participating in the migration of neuronal precursors and in the formation of retinal circuits. Expression of DCX in Müller glia-like cells and in retinal pigment epithelium cells suggests that these cells could be neurogenic in lampreys. The comparison of the spatio-temporal pattern of expression of DCX between lampreys and jawed vertebrates shows conserved characters, such as the expression of DCX in migrating neuronal precursors or in immature ganglion and horizontal cells, but also important differences like the expression of DCX in photoreceptors.

## Author Contributions

BF-L, DR-S, PS-M, AB-I and MCR contributed to the acquisition of experimental data, data analysis/interpretation and drafting of the manuscript. RA contributed to the data analysis/interpretation and critical revision of the article. AB-I and MCR also contributed to the concept/design of the study. All authors have approved the final manuscript.

## Funding

This work was supported by grants from the Spanish Ministry of Science and Education (BFU2004-01080) and Xunta de Galicia (PGIDT04PXIB020003PR; PGIDIT05PXIC2004PN; INCITE08PXIB200063PR; INCITE09ENA200036ES; GPC2014/030). AB-I was supported by an I2C postdoctoral grant from the Xunta de Galicia.

## Conflict of Interest Statement

The authors declare that the research was conducted in the absence of any commercial or financial relationships that could be construed as a potential conflict of interest.

## Abbreviations

CP: Crystalline placode
DCX: Doublecortin
H-E: Hematoxylin-eosine
INbL: Inner neuroblastic layer
INL: Inner nuclear layer
IPL: Inner plexiform layer
OLM: Outer limitans membrane
ONbL: Outer neuroblastic layer
ONL: Outer nuclear layer
Ret: Retina.
